# Suppressed visual looming stimuli are not integrated with auditory looming signals: Evidence from continuous flash suppression

**DOI:** 10.1068/i0678

**Published:** 2015-03-17

**Authors:** Pieter Moors, Hanne Huygelier, Johan Wagemans, Lee de-Wit, Raymond van Ee

**Affiliations:** Laboratory of Experimental Psychology, University of Leuven (KU Leuven), Leuven, Belgium; e-mail: pieter.moors@ppw.kuleuven.be; Laboratory of Experimental Psychology, University of Leuven (KU Leuven), Leuven, Belgium; e-mail: hanne.huygelier@student.kuleuven.be; Laboratory of Experimental Psychology, University of Leuven (KU Leuven), Leuven, Belgium; e-mail: johan.wagemans@psy.kuleuven.be; Laboratory of Experimental Psychology, University of Leuven (KU Leuven), Leuven, Belgium; e-mail: lee.dewit@ppw.kuleuven.be; Laboratory of Experimental Psychology, University of Leuven (KU Leuven), Leuven, Belgium; Department of Biophysics, Donders Institute, Radboud University, Nijmegen, The Netherlands; Department of Brain, Body, & Behavior, Philips Research Laboratories, Eindhoven, The Netherlands; e-mail: raymond.vanee@ppw.kuleuven.be

**Keywords:** binocular rivalry, continuous flash suppression, interocular suppression, audiovisual integration, audiovisual looming, multisensory integration, visual awareness

## Abstract

Previous studies using binocular rivalry have shown that signals in a modality other than the visual can bias dominance durations depending on their congruency with the rivaling stimuli. More recently, studies using continuous flash suppression (CFS) have reported that multisensory integration influences how long visual stimuli remain suppressed. In this study, using CFS, we examined whether the contrast thresholds for detecting visual looming stimuli are influenced by a congruent auditory stimulus. In Experiment 1, we show that a looming visual stimulus can result in lower detection thresholds compared to a static concentric grating, but that auditory tone pips congruent with the looming stimulus did not lower suppression thresholds any further. In Experiments 2, 3, and 4, we again observed no advantage for congruent multisensory stimuli. These results add to our understanding of the conditions under which multisensory integration is possible, and suggest that certain forms of multisensory integration are not evident when the visual stimulus is suppressed from awareness using CFS.

## 1 Introduction

When both eyes are presented with incompatible images, the phenomenological consequence usually is one of binocular rivalry in which the percept alternates between the two images instead of mixing them into one coherent interpretation (Blake & Logothetis, 2002; Tong, Meng, & Blake, 2006). Alternations between rivaling percepts can be modulated by numerous factors, including the contrast (Fox & Rasche, 1969) or spatial frequency (Fahle, 1982) of the stimuli, the allocation of attention (Meng & Tong, 2004; Ooi & He, 1999; Paffen, Alais, & Verstraten, 2006; van Ee, van Dam, & Brouwer, 2005), and stimulus predictability (Chopin & Mamassian, 2012). In addition, it has become clear that nonvisual stimuli can influence the rivalry between visual stimuli, from audition (Alais, van Boxtel, Parker, & van Ee, 2010; Chen, Yeh, & Spence, 2011; Conrad et al., 2013; Conrad, Bartels, Kleiner, & Noppeney, 2010; Guzman-Martinez, Ortega, Grabowecky, Mossbridge, & Suzuki, 2012; Kang & Blake, 2005; van Ee, van Boxtel, Parker, & Alais, 2009), to touch (Lunghi & Alais, 2013; Lunghi, Binda, & Morrone, 2010; Lunghi, Morrone, & Alais, 2014; Lunghi & Morrone, 2013), and even olfaction (Zhou, Zhang, Chen, Wang, & Chen, 2012; Zhou, Jiang, He, & Chen, 2010). Most of these studies have demonstrated that the influence of multisensory stimulation from the auditory modality specifically increases the duration of the already dominant (conscious) stimulus, rather than causing visual perception to switch to a nondominant (unconscious) stimulus (Chen et al., 2011; Conrad et al., 2010; Kang & Blake, 2005). However, some evidence has also been reported for an increased probability of switching to the currently nondominant stimulus when it was congruent with an auditory (Lunghi et al., 2014) or tactile signal (Lunghi & Alais, 2013; Lunghi et al., 2010, 2014; Lunghi & Morrone, 2013).

The question whether multisensory integration can be achieved for a stimulus suppressed from visual awareness through interocular suppression has recently been readdressed in studies in which continuous flash suppression (CFS) was used as the interocular suppression paradigm (Tsuchiya & Koch, 2005; Tsuchiya, Koch, Gilroy, & Blake, 2006). CFS is a binocular rivalry variant in which a dynamic noise pattern (usually consisting of shapes of random size and orientation, called a CFS mask) is presented to one eye. In most implementations, the mask content refreshes every 100 milliseconds (ms) (i.e., at 10 Hz), yielding robust suppression of the stimulus presented to the other eye. CFS provides interesting advantages over the use of regular binocular rivalry for assessing multisensory integration in the absence of visual awareness. The CFS mask is usually dominant at stimulus onset, enabling stricter control over which stimulus dominates in visual awareness at the start of each trial. This provides the opportunity to assess specifically whether the suppressed stimulus is integrated with the stimulus presented in the nonvisual modality.

Studies in which CFS was used as the interocular suppression paradigm have reported evidence that multisensory integration can occur in the absence of awareness (Alsius & Munhall, 2013; Palmer & Ramsey, 2012; Plass, Guzman-Martinez, Ortega, Grabowecky, & Suzuki, 2014; Salomon, Lim, Herbelin, Hesselmann, & Blanke, 2013; Yang & Yeh, 2014; Zhou et al., 2010). Most of these studies have relied on breaking CFS (b-CFS) (a term coined by Stein, Hebart, & Sterzer, 2011, based on a paradigm introduced by Jiang, Costello, & He, 2007) in which a stimulus is suppressed through CFS and gradually increased in contrast until it “breaks through” the CFS mask (i.e., becomes detectable). Upon breakthrough, participants usually have to perform a speeded localization task on the stimulus. Differential suppression times for different stimuli are then attributed to differences in stimulus processing during suppression (e.g., as in Jiang et al., 2007). For example, Zhou et al. (2010) reported that the congruency between an olfactory stimulus (i.e., the smell of a rose or a marker) and a visual stimulus (i.e., an image of a rose or a marker) suppressed by CFS can bias suppression times such that congruent stimuli break through suppression faster than incongruent stimuli. Similarly, Alsius and Munhall (2013) reported that the congruency relation between auditory stimuli and a visual lip-stream sequence suppressed from awareness by CFS modulates suppression times such that the congruent stimulus combination breaks through suppression faster. These findings seem to indicate that multisensory integration can indeed take place in the absence of awareness of one of the modalities (the visual one) and that the supraliminal modality can bias the breakthrough times of the suppressed stimulus. However, these studies have relied on b-CFS, and the validity of this paradigm to assess unconscious processing of the suppressed stimulus has recently been questioned (Stein et al., 2011; Stein & Sterzer, 2014). Because the responses in the b-CFS paradigm rely on the participants being conscious of the stimulus of interest, it is necessary to ensure that the difference in suppression times is driven by a difference in the time at which stimuli break through suppression. That is, different stimuli could break through suppression on average at the same time, yet the critical stimulus manipulation could influence the participants' response time to one of the stimulus classes. This would yield a difference in suppression times that is not attributable to differences in processing during suppression, but rather to postperceptual or decisional factors. To rule this out, a control condition is traditionally used in which the CFS mask and the stimulus are both presented to both eyes (binocular control condition in which no interocular suppression takes place). However, this control condition has been shown to be insufficiently comparable to the CFS condition to infer unconscious processing (Stein et al., 2011). This has led Stein and Sterzer (2014) to argue that b-CFS, as it is currently used, cannot provide evidence for unconscious processing.

### 1.1 The present study

In this study, we set out to reevaluate whether multisensory integration can be achieved between a suppressed visual stimulus and a supraliminal auditory stimulus using a different paradigm than b-CFS. To do so, we tested whether a visual looming stimulus (as previously used by van Ee et al., 2009), while being suppressed by CFS, can be integrated with a concurrently presented tone pip or looming sound, by measuring detection thresholds of the visual looming stimulus in different conditions. As in the previous studies on multisensory integration during interocular suppression, we opted to use CFS because it can ensure that the visual looming stimulus is suppressed at trial onset. To avoid the problems associated with b-CFS, we fixed the presentation time of the stimuli and measured contrast detection thresholds for the visual looming stimulus. Such an accuracy-based measure has been used in previous studies (Kaunitz, Fracasso, Lingnau, & Melcher, 2013; Stein et al., 2011; Tsuchiya et al., 2006; van der Groen, van der Burg, Lunghi, & Alais, 2013; Yang & Blake, 2012) and largely avoids the potential problem of differential response criteria in the classic implementation of b-CFS (Stein et al., 2011). Furthermore, we reasoned that if audiovisual integration indeed takes place in the absence of awareness of the visual looming stimulus, suppression strength in this condition would be lower than in the other conditions (Kang & Blake, 2005). Consequently, the thresholds in this condition would be expected to be lower compared to an incongruent or visual-only condition.

We were interested in testing whether a suppressed visual looming stimulus could be integrated with a supraliminal auditory (looming) stimulus not only because looming is a biologically relevant signal that might be crucial for survival (Bach, Neuhoff, Perrig, & Seifritz, 2009; Fotowat & Gabbiani, 2011; Ghazanfar, Neuhoff, & Logothetis, 2002; Maier, Neuhoff, Logothetis, & Ghazanfar, 2004; Neuhoff, 1998, 2001; Schiff, Caviness, & Gibson, 1962), but also because previous studies already have demonstrated that a looming stimulus dominates perception in binocular rivalry and that attentional allocation to a rhythmically congruent auditory looming signal can boost the attentional effect of holding the looming stimulus in perceptual dominance (Conrad et al., 2013; Parker & Alais, 2007; van Ee et al., 2009). Furthermore, neuropsychological evidence suggests that looming stimuli can be processed in the absence of awareness. That is, extinction due to brain damage has been reported to be less severe for looming stimuli compared to contracting stimuli (Dent & Humphreys, 2011). Lastly, with respect to the neural locus of audiovisual looming integration, a recent fMRI study by Tyll et al. (2013) documented superadditive fMRI responses in visual cortex for multisensory looming signals compared to unisensory signals. Furthermore, the authors reported enhanced functional connectivity between the superior temporal sulcus and lower-level visual areas during multisensory looming stimulation. Since multisensory looming signals appear to be processed at least partially in early visual cortex, this finding prompts the question as to whether CFS completely abolishes the potential for audiovisual looming integration or whether the visual looming signal is sufficiently preserved to allow for the auditory signal to interact with the visual looming signal.

In sum, based on the available evidence on multisensory integration between a suppressed visual stimulus and a suprathreshold stimulus of the nonvisual modality, the present study addressed the extent to which a suppressed visual looming stimulus could be integrated with an auditory (looming) signal. This question was particularly motivated by the biological relevance of looming, the available neuropsychological evidence on audiovisual looming integration in brain-damaged patients as well as the neural locus of fully visible audiovisual looming stimuli. Furthermore, by measuring contrast detection thresholds of the visual looming stimulus in different experimental conditions, we were able to explicitly test whether auditory stimulation directly influenced the strength of the suppressed stimulus. We conducted four experiments to assess whether an auditory (looming) signal influenced contrast detection thresholds of a visual looming stimulus. To preview our results, we obtained no evidence in favor of audiovisual integration of suppressed visual looming stimuli and auditory tone pips (Experiments 1, 2, and 4) or looming sounds (Experiment 3).

## 2 Experiment 1

The goal of Experiment 1 was to assess whether visual looming per se would lead to lower contrast detection thresholds compared to a static concentric grating. Second, we sought to test whether an auditory tone pip could further lower contrast detection thresholds when presented in a rhythmically congruent fashion with the visual looming stimulus. Thus, Experiment 1 consisted of three conditions: visual static, visual looming, and audiovisual looming. On each trial, participants had to indicate whether the target stimulus, initially suppressed from visual awareness through CFS, was presented above or below fixation and contrast detection thresholds were measured using a QUEST staircase procedure (Watson & Pelli, 1983).

### 2.1 Methods

#### 2.1.1 Participants

Seventeen participants took part in this study, two of which were the first authors. All participants had normal hearing and normal or corrected-to-normal vision. The mean age was 22.5 years (*SD* = 1.71). All participants signed an informed consent. All experiments were conducted in line with the ethical principles regarding research with human participants as specified in The Code of Ethics of the World Medical Association (Declaration of Helsinki). The study was approved by the Ethical Committee of the Faculty of Psychology and Educational Sciences (EC FPPW) of the University of Leuven.

#### 2.1.2 Apparatus and stimuli

All experiments were conducted in a dark room. The stimuli were created with PsychoPy (Peirce, 2007, 2009) and presented on two gamma-corrected color cathode ray tube (CRT) monitors (2048 × 1536 resolution for each monitor). Participants viewed a pair of dichoptic displays through a mirror stereoscope at a distance of 125 cm with a chin rest. The refresh rate of the monitor was 60 Hz. A checkerboard pattern was continuously presented to ensure binocular fusion (checker size 0.34°). A fixation cross with (0.6° × 0.6°) was continuously presented in the center of the screen.

In the eye dominance measurement phase, the target stimulus was an arrow (maximal length 4°; maximal height 2°) pointing leftward or rightward which was presented at the center of the screen. A grayscale CFS mask consisting of Mondrian patterns was presented against a uniform gray background at mean luminance (25 cd/m^2^) with a frame size of 5° × 5°. The CFS mask consisted of 150 elements (squares) presented within a range of 3° × 3° with a contrast (root mean square) of 27.5% and refreshed every 0.10 s (i.e. at 10 Hz). The position and size of the mask elements were randomly alternated. The size of the elements ranged from 1° to 2°.

**Figure 1.**
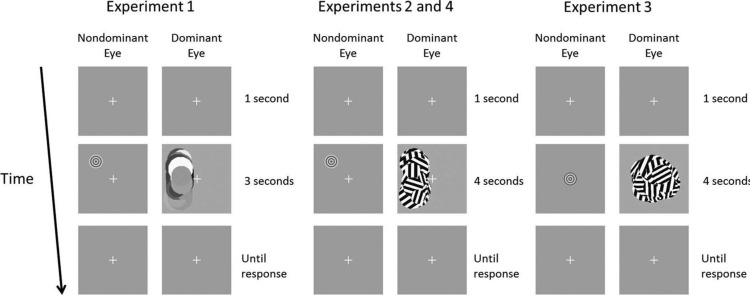
Trial sequence in all the experiments. A trial always started with 1 s of fixation after which the CFS mask and target stimulus (above or below fixation) were presented for 3 (Experiment 1) or 4 s (Experiments 2, 3, and 4). After stimulus presentation, the fixation display was presented until participants made their response.

For the main part of the experiment, visual stimuli were presented on a uniform gray background with a size of 2.5° × 2.5° at mean luminance (see Figure 1 for an example of the trial sequence). The target stimulus consisted of a concentric sine wave grating with a size of 1° and a spatial frequency of 3 cycles per degree. The stimulus was either presented static or with looming motion. The appearance of looming was created by phase shifts. The magnitude of the phase shift was increased exponentially over a 0.8 s period from 0 to 4 Hz and was slowed down according to a cosine decay for 0.2 s such that one looming cycle lasted 1 s (i.e., looming motion at 1 Hz). This implementation of looming motion was based on stimuli used in previous experiments (Parker & Alais, 2007; van Ee et al., 2009). The auditory stimulus was a pure tone of 200 Hz that was presented together with the visual looming stimulus at the peak of each looming cycle for 0.28 s (the amplitude of the tone was modulated such that it peaked after 0.14 s). The CFS mask consisted of 50 circular mask elements alternating randomly in position and size (within a range of 1° and 2°). The mask was refreshed every 0.10 s. The CFS mask was presented in the left half of the visual field (1.5° × 1°; 27.5% contrast, root mean square). The target stimulus was always presented 1.25° left of fixation and either 1.25° above or below fixation.

#### 2.1.3 Procedure and design

Participants read written instructions before the experiment started. They were instructed that they could use the auditory stimulation to perform better on the task. This instruction was given because van Ee et al. (2009) reported that attending the auditory looming signal was critical for observing the multisensory facilitating effect on holding the visual looming stimulus in perceptual dominance. Participants were asked to fixate the fixation cross during the entire experiment. The most important instructions were verbally repeated. First, eye dominance was measured for each participant, based on the technique developed by Yang, Blake, and McDonald (2010). On each trial, an arrow which was gradually ramped up in contrast (100% after 2 s) was presented to one eye while the CFS mask was presented to the other eye, yielding initial perceptual suppression of the arrow. Upon breakthrough of the arrow, participants had to determine the direction of the arrow as fast as possible. In half of the trials, the arrow was presented to the left eye and in the other half to the right eye, for 80 trials in total (40 for each eye). The index for eye dominance was determined by calculating the ratio of the mean suppression time when the arrow was presented in the left eye and the mean suppression time when the arrow was presented in the right eye (Yang et al., 2010). After determining eye dominance, the CFS mask was always presented to the dominant eye during the rest of the experiment. A 15-min break was given after the eye-dominance measurement. The break was necessary to ensure the effectiveness of suppression on participants in the second part of the experiment.

In the second part, the instructions were repeated and—when ready—participants started the experiment. In the visual static condition (VS), only the concentric grating was presented to the nondominant eye. In the visual looming condition (VL), the concentric grating was presented with looming motion. Lastly, in the audiovisual looming condition (AVL), the auditory tone pips were presented concurrently with the visual looming stimulus at the peak of each looming cycle, using a headset. Each condition consisted of 50 trials. On each trial, one of the conditions was randomly selected. A trial started with 1 s of fixation, after which the CFS mask and target stimulus (at the current contrast level of the staircase) were presented for 3 s. After 3 s, both disappeared and the participant had to respond at which spatial position—above or below fixation—the target stimulus had been presented (the spatial location of the target was randomly determined on each trial, but balanced across the experiment). Participants were instructed to guess if they had not seen the target during a trial. A QUEST staircase procedure was used to measure 75% contrast detection thresholds (Watson & Pelli, 1983) for each condition separately. The number of trials necessary for an accurate measurement of the detection threshold was based on a pilot study.

### 2.2 Results

Before subjecting the data to any statistical test, the thresholds were normalized to the mean threshold for each participant (computed across conditions). One participant was excluded from data-analysis due to suppression being too effective. For this participant, the looming stimulus had to be presented at full contrast in all conditions in nearly all trials with performance levels still around chance (50%). In comparison, the average 75% contrast threshold across conditions and participants was 10%.

All analyses were done in a Bayesian framework, relying on Bayes Factors (BF) and 95% credible intervals (CI) on effect sizes. Calculation of BFs was done with the Bayes Factor package (Rouder, Morey, Speckman, & Province, 2012). The models used to analyze the data are conceptually very similar, if not the same, to the classical repeated measures ANOVA. The advantages of statistical inference in a Bayesian compared to a frequentist framework have been elaborately discussed elsewhere (Kruschke, 2010; Kruschke, 2010; Rouder, Speckman, Sun, Morey, & Iverson, 2009; Wagenmakers, 2007). With respect to this study, a major advantage of the use of BFs is that it quantifies the relative evidence for one model compared to another. Thus, and critical for our study, BFs can also be used to quantify the evidence for a null model (containing no effect of condition) compared to a model containing an effect of condition, which is impossible in standard null hypothesis significance testing. We use the classification proposed by Jeffreys (1961) as a guideline for interpreting the BFs (i.e., BFs > 3 or BFs < 1/3 will be considered substantial evidence for the one or the other model). 95% CIs were based on the posterior distribution of the effect size parameter of the model that was estimated (see Rouder et al., 2012, 2009).

**Figure 2.**
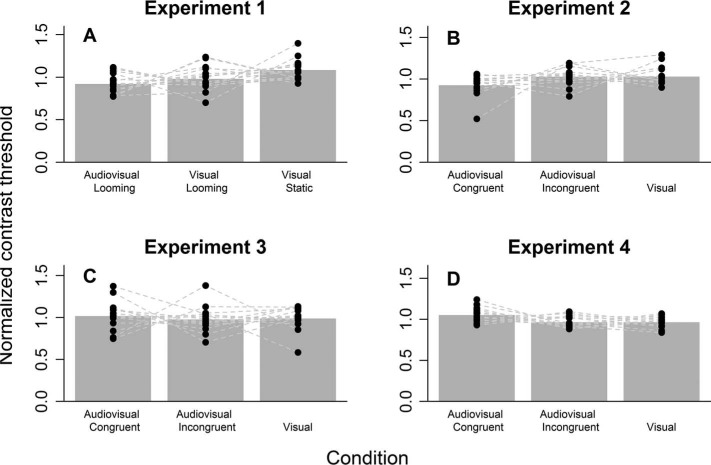
Summary of all four experiments. Bar plots depict the mean normalized contrast threshold for each condition. The dots indicate the normalized contrast thresholds for each participant. Connected dots refer to the same participant.

Figure 2A depicts the mean normalized thresholds and the individual data for each subject. The BF analysis revealed that a condition effect was indeed present in the data (BF = 17). To disentangle the relative contribution of each condition to this effect, different contrasts were computed (summarized in Table 1). From Table 1, it is clear that the condition effect is driven by the difference between the AVL and VS condition. Although the AVL and VL conditions do not differ from each other, their combination does differ from the VS condition. This analysis is complemented by the 95% CIs which do not include zero for the AVL vs. VS and AVL/VL vs. VS contrasts.

**Table 1.** Contrast analysis for Experiment 1.

Contrast	BF (relative to null)	Delta	95% CI
AVL vs. VL	0.41	−0.23	[−0.69; 0.22]
AVL vs. VS	15	−0.79	[−1.36; −0.23]
VL vs. VS	0.88	−0.37	[−0.89; 0.09]
AVL and VL vs. VS	6	−0.65	[−1.21; −0.12]

**Note.** Bayes factors > 3 indicates substantial evidence against the null model. Delta refers to the mean posterior effect size. The 95% CI was calculated for this delta parameter.

### 2.3 Discussion

In Experiment 1, a strong effect was found for the combination of auditory stimulation and looming motion on detection thresholds compared to the static stimulus. However, the results did not indicate that an audiovisual looming stimulus could be detected more easily—when initially suppressed from awareness—than a visual looming stimulus. Comparing both conditions that contained looming motion to the static stimulus indicated that the improvement in detection in the audiovisual looming condition seemed to be mostly driven by the looming motion characteristics of the target stimulus and was not driven by presenting auditory congruent stimulation together with the looming motion.

Because the average contrast at which participants detected the target was quite low (∼10%), a floor effect might have obscured any, potentially bigger effects in a part of our sample. Therefore, we sought to improve the effectiveness of our CFS mask. Recently, some studies have reported evidence on the importance of feature similarity between the CFS mask and the suppressed stimulus to achieve effective suppression (Hong & Blake, 2009; Maehara, Huang, & Hess, 2009; Moors, Wagemans, & de-Wit, 2014; Yang & Blake, 2012). Therefore, we changed the properties of our mask to contain spatial frequency information matching that of the target stimulus.

Stimulus presentation during every trial amounted to 3 s in Experiment 1, and this might have been too short to allow for integration between the visual and auditory stimulus. Therefore, we increased the trial duration in the second experiment. The visual static condition wass excluded, because the results of Experiment 1 indicated that the detection of the target stimulus in the static condition was reliably worse than in the audiovisual looming condition. An incongruent audiovisual condition was added to determine whether any facilitating effect of the audiovisual condition was due to generic auditory stimulation rather than multisensory integration of congruent signals.

## 3 Experiment 2

### 3.1 Methods

#### 3.1.1 Participants

Fourteen of the same participants as in Experiment 1 and the two first authors took part in the second study. The participant that had to be excluded in the analysis of the first experiment was not invited to participate again. Two other participants did not wish to return to participate in the second study. The mean age of participants was 22 years (SD = 1.46). All participants signed an informed consent.

#### 3.1.2 Apparatus and stimuli

The same settings were used as in Experiment 1 for the background, checkerboard pattern, fixation cross, and visual target stimulus. The CFS mask was adjusted to more optimally match the characteristics of the target stimulus. The new mask consisted of 50 circular square wave gratings with a randomly alternating size within a range of 1° and 2° (Figure 1). The spatial frequency of each element was randomly selected within a range of 2–4 cycles/degree and the orientation of each element ranged between 0 and π radians. In the audiovisual congruent condition, the tone pips were presented in the same way as in Experiment 1. In the audiovisual incongruent condition, the tone pips were rhythmically incongruent with the visual looming stimulus and presented at 0.8 Hz instead of 1 Hz as in the audiovisual congruent condition. All other settings of the experiment were kept the same as in Experiment 1.

#### 3.1.3 Procedure and design

Participants signed an informed consent and read written instructions. Since the majority of participants participated in Experiment 1, eye dominance did not need to be measured again for these participants. The visual looming (VL) and audiovisual congruent (AVC) conditions were the same as in Experiment 1. In the audiovisual incongruent (AVIC) condition, the tone pips were presented in a rhythmically incongruent fashion with the visual looming stimulus (i.e., at 0.8 Hz instead of 1 Hz). At the start of each trial, a fixation cross was presented for 1 s after which the target stimulus and CFS mask were presented for 4 s. After stimulus presentation, participants responded whether they saw the target stimulus above or below fixation. Target position was again randomly determined on each trial, but balanced across the experiment. The three different conditions—visual, audiovisual congruent and audiovisual incongruent— were presented in a random order for 50 trials per condition. 75% contrast detection thresholds were measured again using a QUEST staircase procedure (Watson & Pelli, 1983).

### 3.2 Results

Since the participants were partly the same as in Experiment 1, we could explicitly test whether the new CFS mask improved suppression compared to the one in Experiment 1. An improvement in average contrast was indeed observed (BF = 5; posterior mean effect size 0.51; 95% CI = [0.11; 0.91]). Figure 2B depicts the results of Experiment 2. The data were again analyzed using the normalized contrast thresholds (relative to the mean of each participant across conditions). One subject was not included in the analysis because the mean contrast threshold deviated more than three standard deviations from the mean contrast threshold across all subjects. The BF analysis indicated no convincing evidence (BF = 2.11) for an effect of condition on the normalized contrast thresholds. The follow-up contrast analysis is summarized in Table 2. It is clear that the evidence for an effect of the AVC condition versus the other conditions is indecisive, i.e., both the null model and the model that indicates a difference between conditions are favored equally. Moreover, the results were strongly influenced by one participant with a normalized contrast threshold of approximately 0.5 in the AVL condition. Exclusion of this influential data point reversed the direction of the BF to weak evidence for the null model (BF = 0.68).

**Table 2.** Contrast analysis for Experiment 2.

Contrast	BF (relative to null)	Delta	95% CI
Including influential data point			
AVC vs. AVIC	1	−0.40	[−0.91; 0.08]
AVC vs. VL	1	−0.40	[−0.92; 0.1]
AVIC vs. VL	0.26	−0.02	[−0.48; 0.44]
AVC vs. AVIC and VL	1.29	−0.45	[−.97; 0.05]
Excluding influential data point			
AVC vs. AVIC	0.70	−0.35	[−0.88; 0.14]
AVC vs. VL	0.85	−0.38	[−0.91; 0.13]
AVIC vs. VL	0.27	0.02	[−0.47; 0.50]
AVC vs. AVIC and VL	1.60	−0.50	[−1.05; 0.02]

### 3.3 Discussion

In Experiment 2, again, no support was found for the hypothesis that the detection of the visual looming target would improve when presented in combination with congruent sound. In sum, in a sample nearly the same as in Experiment 1, we obtained, with an improved design, no evidence for audiovisual looming integration in the context of a subliminal visual looming stimulus. The estimates for the effect sizes for a difference between the audiovisual congruent condition and one of the other conditions were about 0.40, generally considered to be a moderate effect according to the classification proposed by Cohen (1977). If we ignore the Proteus phenomenon (Button et al., 2013) and consider this an accurate estimate of the true effect, a power analysis for an effect size of 0.40 indicates that we should at least quadruple our sample to achieve 90% power.

Instead of quadrupling our sample, we decided to redesign our experiment such that, if multisensory integration is driving the direction of the differences observed in Experiments 1 and 2, these differences should be more pronounced in this experiment or, at least, the same. That is, van Ee et al. (2009) observed an attentional benefit of multisensory stimulation when the looming pattern was presented at fixation (similarly for the looming bias observed in Parker & Alais, 2007). Therefore, we presented the looming stimulus at fixation in Experiment 3. Furthermore, although tone pips were argued to be equally effective in van Ee et al. (2009), we implemented a continuous looming sound to increase the (in)congruency of the auditory and visual signals. That is, in Experiment 3, at every moment in time for the audiovisual conditions, either congruent or incongruent auditory signals were presented together with the visual signal. Last, to ensure that our effects were not specific to our previous sample of participants, we collected data from a new sample of participants.

## 4 Experiment 3

### 4.1 Methods

#### 4.1.1 Participants

Sixteen new observers and one of the first authors participated in the experiment. All new observers participated in return for course credit. The mean age was 19 years (SD = 2.06). All participants signed an informed consent.

#### 4.1.2 Apparatus and stimuli

The visual target stimulus and CFS mask were identical to those in Experiment 2, but they were now presented at fixation. Instead of a tone pip, the auditory stimulus was a looming sound in the current experiment. A pure tone of 200 Hz was presented, periodically increasing and decreasing in amplitude. The increase in amplitude coincided with the phase-shifts of the visual looming motion (1 Hz) in the audiovisual congruent condition. In the audiovisual incongruent condition the frequency of the looming sound was 0.87 Hz. All other settings of the experiment were identical to Experiment 2.

#### 4.1.3 Procedure and design

The procedure was the same as in Experiments 1 and 2, except for the task participants had to perform. Since the target stimulus was always presented at fixation, participants had to indicate on each trial whether they had seen the target stimulus or not (yes/no classification) (Figure 1). The experiment consisted of the three same conditions as in Experiment 2 (visual looming, audiovisual congruent, and audiovisual incongruent). Catch trials (in which no target stimulus was presented) were included for all three conditions such that the absence or presence of sound would not be predictive for target presence. For each condition, we again measured contrast thresholds through a QUEST staircase procedure, for 50 trials per staircase. Fifty catch trials were also included and at each trial it was determined randomly whether an “(in)congruent” sound would be presented during the catch trial or not. Thus, participants performed 200 trials in total. The different conditions were again randomized across trials.

### 4.2 Results

A mean accuracy level of 75% on the catch trials was the cutoff to include data. Two participants did not meet this criterion (8% and 63% accuracy). The mean accuracy of participants included in further analysis was 91.5% (SD = 7.3).

Figure 2C depicts the results of Experiment 3. The BF analysis was again done on the normalized contrast thresholds. As is already apparent from Figure 2C, the omnibus analysis did not indicate evidence for an effect of condition (BF = 0.20). Table 3 summarizes the contrast analysis. Here again, no evidence for an effect is found. To the contrary, the BFs for the omnibus analysis and contrasts indicate strong evidence in favor of the null model (i.e., all BFs < 0.33). Moreover, the direction of the differences between conditions seems to have reversed in Experiment 3 compared to Experiment 2. That is, numerically, the audiovisual congruent condition now had the highest mean normalized contrast detection threshold.

**Table 3.** Contrast analysis for Experiment 3.

Contrast	BF (relative to null)	Delta	95% CI
AVC vs. AVIC	0.30	0.11	[−0.35; 0.58]
AVC vs. VL	0.28	0.09	[−0.37; 0.56]
AVIC vs. VL	0.27	−0.04	[−0.51; 0.42]
AVC vs. AVIC and VL	0.30	0.11	[−0.34; 0.57]

### 4.3 Discussion

In Experiment 3, we tested a new sample of participants with a further refined design to maximize our chances of finding an effect of multisensory integration on detecting a suppressed visual looming stimulus. That is, we presented the looming stimulus at fixation and we used a continuously looming sound that was either congruent or incongruent with the visual looming stimulus. Again, no effects of (synchronous) auditory stimulation were observed. All BFs indicated strong evidence for the absence of an effect for any comparison between conditions that was considered.

To increase our confidence in this null effect, we decided to run Experiment 2 again on another sample of participants. We reasoned that if a true effect underlay the direction of the differences in Experiment 2, we should at least be able to replicate this pattern in a new sample of subjects, albeit the sample size not providing sufficient power. In contrast, if a null effect underlay the data of Experiment 2, we would expect that this new sample might show differences between conditions in different directions compared to Experiment 2. Crucially, however, analyzing the sample of Experiment 2 together with the new sample should cancel out any differences observed in both samples separately, if we are dealing with a null effect.

## 5 Experiment 4

### 5.1 Methods

#### 5.1.1 Participants

Fifteen paid observers participated in the experiment. Their age ranged between 18 and 30 years. All observers provided informed consent before the start of the experiment.

#### 5.1.2 Apparatus and stimuli

The experimental setup and stimuli were identical to Experiment 2.

#### 5.1.3 Procedure and design

The procedure and design were exactly the same as in Experiment 2.

### 5.2 Results and Discussion

**Figure 3.**
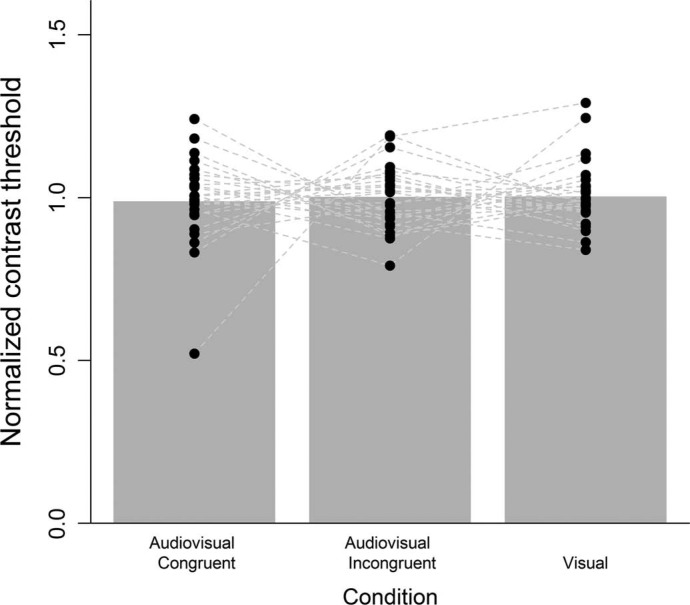
Mean normalized contrast thresholds collapsed for Experiments 2 and 4. The dots indicate the normalized contrast thresholds for each participant. Connected dots refer to the same participant.

Figure 2D depicts the results of Experiment 4. The data were again analyzed using the normalized contrast thresholds. Two participants were excluded from the analysis because at least one of their contrast thresholds estimated by the QUEST procedure exceeded the maximum contrast level. The BF analysis now indicated strong evidence in favor of an effect for condition (BF = 6). However, comparing these results with those obtained in Experiment 2, it becomes clear that the direction of the differences has now reversed. Thus, in a second analysis, we collapsed the data of Experiment 2 and Experiment 4 (Figure 3). The BF analysis now indicated substantial evidence for the null model (BF = 8). Thus, collecting additional data for Experiment 2 revealed that in the new sample an effect of condition was present (and stronger compared to Experiment 2, yet in a different direction), but an analysis of the aggregate data indicated substantial evidence for no differences between conditions.

## 6 General Discussion

We examined whether an auditory signal can be integrated with a visual stimulus suppressed from awareness through Continuous Flash Suppression (CFS). To this end, we used a visual looming stimulus and concurrently presented tone pips (Experiments 1, 2, and 4) or a continuous looming sound (Experiment 3) and measured contrast detection thresholds of the visual looming stimulus. Multisensory integration between the supraliminal auditory stimulus and subliminal visual stimulus should lead to lower contrast detection thresholds in the congruent audiovisual condition compared to a unisensory visual condition or a multisensory condition in which the auditory stimulus was incongruent with the visual looming stimulus. In other words, due to multisensory integration, the strength of the representation of the suppressed visual stimulus would increase relative to the other conditions, effectively lowering suppression strength of the CFS mask and therefore requiring lower contrast to achieve the same performance. Across four experiments, we obtained no evidence for audiovisual integration during interocular suppression. That is, the results were either equally supportive for either an effect of condition or no effect of condition (Experiments 1 and 2) or provided substantial evidence for the null model of no condition effect (Experiment 3, and Experiments 2 and 4 combined). Furthermore, in the contrast analyses, we never obtained convincing evidence for contrast thresholds being lower in the audiovisual congruent condition compared to the audiovisual incongruent or visual-only conditions. Based on these results, the most parsimonious conclusion seems to be that, for the stimulus combinations we have used, integration between an auditory (looming) signal and a visual looming stimulus cannot be achieved in the absence of awareness of the visual stimulus (induced through interocular suppression), or at least, cannot bias breaking suppression in a manner consistent with predictions based on multisensory integration.

Our results thus seem to stand in apparent contrast with other studies using CFS to assess multisensory integration in the absence of awareness (Alsius & Munhall, 2013; Palmer & Ramsey, 2012; Plass et al., 2014; Salomon et al., 2013; Yang & Yeh, 2014; Zhou et al., 2010). The question thus remains as to why other studies have found an effect of multisensory integration for visual stimuli suppressed from awareness, when none was found here. Obviously, we are not in a position to resolve this apparent inconsistency immediately, but it is worth noting a number of the core differences between different studies, particularly in terms of the types of methods and stimuli used.

Although the CFS paradigm provides unique opportunities to assess whether multisensory integration can occur in the absence of visual awareness (Deroy, Chen, & Spence, 2014), previous studies have often relied on breaking CFS (b-CFS) (Alsius & Munhall, 2013; Salomon et al., 2013; Yang & Yeh, 2014; Zhou et al., 2010). As already highlighted in the Introduction, this approach is not without its critiques (Stein et al., 2011; Stein & Sterzer, 2014, see also a recent review by Gayet, Van Der Stigchel, & Paffen, 2014), one particular concern being that the observed difference in suppression time between conditions cannot be unambiguously attributed to processing differences during suppression. It might thus be that the absence of an effect in the current study reflects the use of a more stringent measure, being the contrast threshold at which something can be detected, rather than the time taken for a stimulus to break through suppression. Nevertheless, not all of these studies have relied on b-CFS, so this does not provide a full explanation for the discrepancy between the current study and previous studies.

It is interesting to note that both studies that have not used b-CFS to study multisensory integration for a suppressed visual stimulus were on the subject of audiovisual speech integration. For example, in the study of Palmer and Ramsey (2012) two visual lip-stream sequences were presented while suppressed through CFS and concurrently an auditory stream was presented, congruent with one of the visual lip-stream sequences. After presenting this sequence, the authors probed whether participants had allocated their attention to the spatial location at which the visual lip-stream sequence was either congruent or incongruent with the auditory stream. They did so by presenting a near-threshold Gabor patch in one of the two locations, of which participants had to indicate the location and orientation. The results indicated that participants performed better on valid cue-target trials. Palmer and Ramsey (2012) argued that to be able to do this, the congruency relation between visual and auditory input has to be extracted, presumably through multisensory integration of both signals. A different study showed that participants respond faster to spoken words when a concurrently presented face suppressed by CFS articulates that word compared to when the suppressed face articulates a different word (Plass et al., 2014).

Although our interest in testing whether auditory (looming) stimuli could be integrated with suppressed visual looming stimuli was partly motivated by the ecological and potentially evolutionary relevance of looming stimuli, it seems to be the case that, based on the results of these two studies, visual stimuli that are more naturalistic and potentially more relevant (ecologically and evolutionary) do seem to be integrated with supraliminal auditory stimuli. Indeed, the naturalistic structure of a stimulus has been shown to be an important determinant of audiovisual integration in binocular rivalry (Conrad et al., 2013). Furthermore, it should be noted that audiovisual speech stimuli provide more constraints as to which part of the auditory input should match which part of the visual input, which might be beneficial for integration when one of the inputs is rendered unconscious. Lastly, the results of these two studies particularly pertain to attentional orienting or response preparation in function of integrating subliminal visual and supraliminal auditory input. This type of integration might as well happen for the stimulus used in this study. Yet it could be that, due to the fact that the site of interocular suppression is usually located fairly early in the visual processing hierarchy (Blake & Logothetis, 2002; Logothetis, 1998), the representation of the suppressed stimulus is such that the synergistic effects observed for visible looming stimuli fail to play out in this situation (Cappe, Thelen, Romei, Thut, & Murray, 2012; Tyll et al., 2013).

If we broaden the scope of the discussion to also include studies that used regular binocular rivalry, it is interesting to note that most studies on audiovisual interactions in binocular rivalry observed an effect of the auditory stimulus that was restricted to an extension of dominance periods for the congruent visual stimulus (Chen et al., 2011; Conrad et al., 2010; Kang & Blake, 2005). In that respect, this study could be said to reveal a similar pattern, since there is no effect of auditory stimuli on contrast detection thresholds of suppressed visual looming stimuli. A remarkably different pattern emerges, however, if one considers the interaction between tactile and visual stimuli during binocular rivalry. Here, tactile stimuli do not only increase dominance durations of congruent visual stimuli, but also shorten suppression durations of these congruent visual stimuli (Lunghi & Alais, 2013; Lunghi et al., 2010, 2014; Lunghi & Morrone, 2013). These findings seem to indicate a relatively early phase in the processing hierarchy at which these signals already interact, not only because the tactile stimuli can influence suppression durations, but also because these studies have shown that these effects are tuned to both orientation (Lunghi & Alais, 2013) and spatial frequency (Lunghi et al., 2010). On a speculative note, the difference between audiovisual and tactile–visual integration in binocular rivalry might be related to the level at which these stimuli are integrated. That is, neural processing related to an interocularly suppressed stimulus is mostly restricted to early visual areas (Blake & Logothetis, 2002; Logothetis, 1998). Given that evidence for integration between a suppressed visual stimulus and a tactile but not auditory stimulus is found, this could lead one to hypothesize that tactile-visual integration is possible in early visual areas (at least for the stimuli used in these studies) but that audiovisual integration requires a contribution from higher areas. Indeed, studies have consistently reported an important role for the superior temporal sulcus in audiovisual integration (Stevenson, Geoghegan, & James, 2007; Stevenson & James, 2009; Tyll et al., 2013). Suppressing a visual stimulus through interocular suppression might thus block the feedforward progression of visual input to a stage in the system where audiovisual integration can exert its synergistic effects.

A notable exception to this general pattern is the recent study of Lunghi et al. (2014). In this study, the authors demonstrated not only an increased probability of maintaining the current percept when it was congruent with an auditory or tactile stimulus, but also an increased probability of switching to the other stimulus when the current percept was incongruent with the tactile or auditory stimulus. As such, Lunghi et al. (2014) provided evidence that both auditory and tactile stimuli can be integrated with suppressed visual stimuli during binocular rivalry. It should be noted, however, that this study relied on temporal frequency rivalry (Alais & Parker, 2012). Although temporal frequency rivalry resembles spatial rivalry with respect to dominance duration distributions and alternation rates, the mechanisms through which it acts might as well allow for integration between a supraliminal auditory or tactile stimulus and a suppressed visual stimulus whereas spatial rivalry might not. Lastly, compared to our study, Lunghi et al. (2014) tracked dominance durations, whereas we were interested in potential integration at the first stage of suppression. Taking together these findings, one might thus speculate that integration between a supraliminal auditory or tactile stimulus and suppressed visual stimulus only plays out in the later phases of stimulus presentation, after a few alternations between stimuli have occurred.

## 7 Conclusion

This study sought to address whether multisensory integration between an auditory stimulus and a visual looming stimulus can be achieved when the looming stimulus is presented in the absence of awareness induced through CFS. In four experiments, contrast detection thresholds of the visual looming stimulus were measured in a static (Experiment 1), visual looming (Experiments 1, 2, 3, and 4), audiovisual congruent (Experiments 1, 2, 3, and 4), and audiovisual incongruent (Experiments 2, 3 and 4) condition. The accumulated evidence from these four studies suggests that congruent audiovisual signals are not able to reduce the contrast detection threshold at which visual stimuli can be detected. Thus, for audiovisual looming stimuli, no evidence for multisensory integration in the absence of visual awareness was found. Although these results are in line with the general pattern of results on audiovisual interactions in binocular rivalry (but see Lunghi et al., 2014; Palmer & Ramsey, 2012; Plass et al., 2014), it is currently unclear whether differences with previous studies reflect methodological differences in the measurement techniques used (contrast detection thresholds, b-CFS, or priming), in the modalities tested (auditory vs. tactile) or the types of stimuli used (abstract vs. naturalistic).
